# 术中误吸

**DOI:** 10.3779/j.issn.1009-3419.2020.101.22

**Published:** 2020-05-20

**Authors:** 艺耀 崔, 永 崔

**Affiliations:** 100050 北京，首都医科大学附属北京友谊医院胸外科 Department of Thoracic Surgery, Beijing Friendship Hospital Affiliated to Capital Medical University, Beijing 100050, China

**Keywords:** 术中误吸, 肺肿瘤, 胃酸, 胃蛋白酶, Intraoperative aspiration, Lung neoplasms, Gastric acid, Pepsin

## Abstract

术中误吸是外科手术中常见的肺部并发症，麻醉和体位是导致术中误吸的主要因素。近年来，围手术期肺保护已受到外科和麻醉医师的广泛关注，如何加速术后康复进程，减少相关并发症发生，显著改善患者预后已成为当前外科治疗的主要目标。本文将以术中误吸为重点，从解剖、病理生理、表现、诊断、处理和预防等方面展开综述。

## Abstract (en)

Intraoperative aspiration is a common pulmonary complication in the surgery, anesthesia and position were main factors leading to the operative aspiration. In recent years, perioperative lung protection has attracted wide attention of thoracic surgeons and anesthetist; how to accelerate the process of postoperative rehabilitation, reduce the incidence of related complications and significantly improve the prognosis of patients, these have become a chief goal of surgical treatment. This article will center on operative aspiration and summarize it from anatomy, pathophysiology, manifestation, diagnosis, treatment and prevention.

误吸指口咽部异物或胃反流入口部的胃内容物，经咽喉部进入下呼吸道的过程；发生在手术操作过程中的误吸则称为术中误吸。误吸在危重症患者和全麻患者中的发生率从45%到89%不等^[1, 2]^。麻醉总体上是安全的，但麻醉相关呼吸道误吸则可能是致命的，Olsen等^[3]^回顾性地分析了5年中85, 594例的麻醉患者，其中25例发生误吸，均为全麻。McCaul等^[4]^发现35%的术中误吸主要发生在侧卧位，其胸外科术中误吸的发生频率是其他手术的3倍^[5]^。随着当前肺癌发病率的升高，不论是肺段切除还是肺叶切除，手术仍是治疗肺癌的主要方法^[6, 7]^；同时，随着加速康复外科(enhanced recovery after surgery, ERAS)理念的提出，加速术后康复进程，减少相关并发症发生，显著改善患者预后已成为当前外科治疗的目标。因此，深入了解及其正确识别、处理和预防术中误吸是非常必要的。

## 1 误吸认识历史和相关概念变迁

1848年，Simpson^[8]^首次报道了一位氯仿麻醉下的15岁女孩在行脚趾甲去除术过程中突发缺氧、紫绀，最后死亡的病例，Simpson把这次意外归结为误吸。1946年，Mendelson^[9]^观察到全麻下因误吸死亡的产妇比正常分娩高，经系统总结后发现麻醉是导致产妇误吸的主要因素，发生率约为1.5‰；同时，Mendelson通过分析产妇误吸后的临床症状，提出产妇在手术分娩前需禁食，麻醉前需排空胃和碱化胃液的临床实践，从而使产妇的死亡率显著下降。麻醉相关性误吸逐渐进入外科医师和麻醉医生的关注范围。21世纪，随着ERAS的提出和发展，围手术期肺保护再次引起外科医师的广泛关注。

误吸相关性肺炎(aspiration pneumonitis)主要为无菌性胃内容物进入下呼吸道而导致的肺部化学性损伤，也称化学性肺炎(chemical pneumonitis)或Mendelson’s综合征^[9]^；吸入性肺炎(aspiration pneumonia)则为误吸了定植于口咽部的致病菌及其分泌物所致的肺部感染性疾病，所以又称感染性肺炎(infectious pneumonia)^[10]^。虽然pneumonitis和pneumonia在中文都可译为肺炎，但两者所表达意义和内涵并不一样。前者主要是机械性、化学性、物理性、免疫性和感染性等所致的炎症，而后者则为炎症疾病状态。

## 2 误吸相关解剖结构

人体在直立、清醒状态下很少发生误吸，这与很多解剖生理保护机制有关，如食管上括约肌、食管运动、胃-食管交界区(食管下括约肌和隔肌脚)、胃排空、清除反射(食管-食管上括约肌收缩反射、喉-食管上括约肌收缩反射和咽-食管上括约肌收缩反射)、声带关闭反射(食管声门反射、喉声门反射和咽收肌反射)等^[11-13]^。而食管下括约肌、食管上括约肌以及咽喉部反射作用最为重要^[14-16]^。

### 2.1 食管下括约肌(lower esophageal sphincter, LES)

这是胃反流的主要“屏障”压力，为食管下缘与胃底的锐角，膈肌右脚在食管的腹部周围形成吊索，收缩时形成该括约肌，胃内压与LES压力差决定了胃内容物的反流。

各种增加腹内压从而增加胃内压的病理情况以及胃-食管交界处异常均可增加反流。一些药物可改变LES压力，如止吐药、胆碱能药、琥珀酰胆碱药和抗酸药等能增加LES压力；而抗胆碱药、硫喷妥钠、阿片类药物和吸入性麻醉剂等则可降低LES压力。非去极化肌肉松弛剂、异丙酚和H_2_阻滞剂对LES压力无影响。环状软骨压力和喉罩通气道的使用也能降低LES^[17, 18]^。

### 2.2 食管上括约肌(upper esophageal sphincter, UES)

主要由咽喉部的两个下收缩肌之一的咽环肌形成，它从环状弓的一边延伸到另一边，环绕着咽部。意识清醒情况下关闭食管上部, 但在正常睡眠中和麻醉期间，可处于开放状态^[19]^。

麻醉中，UES的张力会发生改变，压力从40 mmHg下降到10 mmHg以下，尤其是在使用了麻醉诱导剂、苯二氮卓类药物、气体性麻醉剂和非去极化肌肉松弛剂之后下降更为明显。手术完成时残余的神经肌肉阻滞剂也会对UES产生影响，以此增加术后肺部误吸的风险。

### 2.3 喉部反射

正常情况下，人体可通过喉部和气道的反射活动自行清除少量误吸物。反射主要有以下四种：呼吸暂停伴喉痉挛、咳嗽、用力呼气和痉挛性喘息^[20]^。气管和喉在引发上述反射时比支气管强烈，反流引发的咳嗽也可发生在食道。麻醉期时，上述反射都会受到抑制，尤其是全麻并使用肌松药时；此外，意识障碍的患者中，上述反射的敏感性也会显著下降^[21, 22]^。

## 3 误吸的病生及病理改变

### 3.1 胃酸

动物实验发现，大量胃酸进入肺部，可发生显著的肺损伤和炎症反应；如果量小则症状轻微，甚至很难检测到；而反复的胃酸刺激，终可发生肺纤维化^[23]^。胃酸对肺部的损伤早期为酸性物质直接刺激辣椒素敏感性神经元和胃酸对气道上皮的直接腐蚀作用，可有肺不张、支气管周围出血、肺水肿和支气管黏膜上皮细胞的变性，如胃酸能快速去除，则上述改变可够逐渐恢复正常；4 h-6 h后出现急性炎症反应(中性粒细胞为主)^[24]^，伴有肺泡腔内充满白细胞和纤维蛋白；如损伤持续存在，则可导致肺微血管完整性丧失，体液和蛋白质外渗到气道和肺泡，从而增加气道阻力和抑制氧气扩散，使呼吸功增加；此外，水肿液含有直接干扰肺泡表面活性物质的血浆蛋白，最终使得呼吸做功大大增加，48 h后可见肺透明膜形成，伴严重肺水肿、肺泡出血和实变^[25, 26]^。动物实验结果表明，通过冲洗去除抑制性肺泡表面活性物质的血浆蛋白后，使用外源性表面活性物质治疗可显著改善肺的通气功能^[27, 28]^。

同时，炎症因子也发挥作用，如肿瘤坏死因子-α (Tumor necrosis factor-α, TNF-α)、白介素-8 (Interleukin-8, IL-8)、巨噬细胞炎症蛋白-2和中性粒细胞趋化因子-1^[27, 28]^；还有白细胞来源的氧化剂和蛋白酶也参与其中。此外，类花生酸通过与上述炎症因子协同，促进中性粒细胞的浸润和活化，也发挥了重要作用^[29]^，而补体激活则参与全身反应^[30, 31]^。

### 3.2 胃内食物

表现为典型的双峰效应^[32]^，4 h-6 h为早期急性中性粒细胞炎症反应，此后的48 h，单核细胞反应逐渐达到高峰，并开始出现早期肉芽肿的形成。单核细胞趋化蛋白-1可由多种细胞产生，如肺血管内皮细胞和肺泡2型上皮细胞，在促进炎性肉芽肿的形成和发展中具有重要作^[33, 34]^。

### 3.3 胃酸合并食物

动物实验结果表明，胃酸合并食物所导致的急性肺损伤比单一的胃酸或是胃内食物都要严重。通过分析支气管肺泡灌洗液中的白蛋白水平(表明肺泡-毛细血管膜完整性的丧失)发现，蛋白升高水平与肺损伤的严重程度具有协同作用；同时，动脉氧合显著下降，尤其是24 h后，其PaO_2_/FIO_2_的比值符合临床急性呼吸窘迫综合征(acute respiratory distress syndrome, ARDS)的诊断标准^[35]^。

误吸胃酸合并食物的炎症反应程度取决于第一成分(胃酸)和第二成分(食物颗粒)之间的时间延迟。胃酸导致急性肺损伤后8 h内同时误吸胃内食物颗粒比24 h误吸食物颗粒所产生的严重程度高，也就是说，误吸胃酸合并胃内食物所致的急性肺损伤在4 h内可到达最大炎性反应和最大肺组织损伤程度^[36, 37]^。同时，中性粒细胞趋化因子-1、单核细胞趋化蛋白-1、白介素-10等细胞因子在此时间内也显著升高，尤其是白介素-10在6 h和24 h的不同浓度水平，可对两个时间点的肺损伤程度做出预测^[38, 39]^。

以上结果主要来源于动物实验，不同细胞因子的变化在急性肺损伤和ARDS中发挥了重要作用，这也是今后临床基础研究的方向。

## 4 误吸后并发症发生的原因及其机制

误吸的发生在普通人群中很常见，通过闪烁扫描成像可在健康人群(如睡眠时)中的肺内检测到胃内容物^[40-42]^，但健康人群很少发生肺部并发症，表明误吸所致的肺损伤是由多因素决定的，如意识水平、神经功能状态、口咽运动、咳嗽反射和既往是否存在胃食管反流病(gastroesophageal reflux disease, GERD)等^[43, 44]^。

并发症的发生主要取决于误吸物的量、成分以及宿主相关防御机制。如果量小且温和，同时宿主防御功能正常，一般不会出现相关并发症；反之则容易发生^[45]^。所以，并发症的发生主要跟以下2项有关：(1)下呼吸道防御保护机能受损，如声门关闭、咳嗽反射或其他廓清机制障碍；(2)存在损害下呼吸道的误吸物，如如胃酸、细菌、液体或食物颗粒等。任何减弱条件1和增强条件2的原因都可使并发症发生，主要包括：(1)意识障碍：镇静、麻醉、酒精中毒和药物过量；(2)气道防御功能下降：声带麻痹、气管内插管；(3)吞咽功能障碍：神经性疾病所致，如卒中、多发性硬化、帕金森综合征、痴呆；上呼吸消化道改变，如肿瘤、手术、放疗；食管疾病，如肿瘤、手术、运动障碍、气管食管瘘；(4)胃食管反流病；(5)反复呕吐^[46-48]^。

全麻后导致误吸增加的因素一是全麻使患者意识水平下降，二是全麻使患者保护性反射消失，主动性呕吐减少和被动反流增加^[49-51]^。它可发生在麻醉实施的任何过程中，如麻醉诱导期，维持期和恢复期。因此，麻醉时相是要需要考虑的重要因素。有研究发现，麻醉诱导期即可发生误吸，这需要进行气道紧急处理；发生在麻醉维持期的误吸主要与麻醉不足和无保护性气道有关；麻醉恢复期则与患者体位有关，尤其是仰卧位更易发生^[52]^。除麻醉时相外，还存有其他危险因素：(1)患者自身因素：如饱食(急诊手术)、肠梗阻、腹痛、糖尿病或创伤所致的胃排空障碍等；(2)使用阿片类等可影响胃排空的相关药物；(3)气道管理、麻醉技术以及患者体位等，此外还有麻醉的深度。

## 5 误吸的分类

(1) 根据误吸物的不同，可有3种临床综合征，化学性肺炎、细菌感染性肺炎和气道梗阻。3种类型可有重叠，如化学性肺炎伴有细菌感染等^[53]^。(2)根据误吸物损伤部位可分为2大类。A气道综合征：声带功能异常，慢性咳嗽，支气管收缩(哮喘加重、支气管痉挛)，支气管扩张，弥漫性吸入性细支气管炎，闭塞性细支气管炎综合征；B肺实质综合征：间质性肺病，化学性肺炎，细菌性肺炎(社区获得性肺炎、医院获得性肺炎、吸入性肺炎)。(3)根据是否合并感染分为误吸相关感染性肺炎和误吸相关非感染性肺炎。(4)根据误吸物吸入量分为大量误吸和微量误吸^[48, 54-57]^。

## 6 临床表现

术中突然发生的显著呼吸困难和逐渐加重的低氧血症，患者肢端出现发绀，听诊可闻及肺部弥漫性破裂音，胸片可见下叶后段和上叶后段的侵润性改变，此时应警惕误吸的发生，如误吸物长时的存留，后期可伴有体温升高和血象的异常^[10, 58-60]^，甚至可发展为急性肺损伤或ARDS，进而延长住院时间并增加死亡率^[61]^。

## 7 诊断

麻醉插管或是手术过程中，如直接观察到口咽部反流出胃内容物或是气管镜看到反流物直接进入气管或是下呼吸道，即可做出误吸的诊断，诊断并无困难。但是，对于微量误吸，肉眼很难做出判断，如果存在持续低氧血症、氧饱和度低、高气道阻力、支气管痉挛以及插管后的异常呼吸音等，麻醉医生应警惕患者发生了微量误吸的可能。如果患者术后持续性低氧血症、发绀、肺炎、甚至ARDS，也应考虑术中可能发生了误吸。然而，临床体征对于诊断并无特异性，所以，长期以来，快速简便、敏感性和特异性都很高的微量误吸诊断方法成为了有效控制误吸及其并发症发生的关键。

### 7.1 纤维支气管内镜

由于其可直接观察到气道中的误吸物，可弯曲性纤维支气管内镜一度成为误吸诊断方法，但该方法要求操作者的水平高，受操作者水平的影响较大，结果的变异度也较大，就算是由操作熟练者进行判断，其敏感性也仅有30%，因此，该方法直至今日并没有完全应用于临床^[62]^。

### 7.2 pH测量计

通过测定胃外pH的数值，可间接反映胃酸及内容物的反流，当前较常用的有食管动态pH监测，食管内pH监测，无线胶囊pH监测，多通道管腔内阻抗pH技术，食管压力测定等^[63-65]^。上述方法各有优缺点，敏感性和特异性也存在差异^[66, 67]^，它们在诊断胃食管反流疾病中占有主要地位，并未在术中使用。

### 7.3 胃蛋白酶检测

20世纪60年代，Goldberg等^[68, 69]^发现胃食管返流疾病的很多食管外临床表现与胃蛋白酶有关。Hayat等^[70]^研究发现，唾液中的胃蛋白酶浓度在鉴别反流误吸的患者中具有一定的灵敏性和特异性，当唾液中胃蛋白酶的浓度大于16 ng/mL时，其灵敏度和特异度分别为78.6%和64.9%，而浓度 > 210 ng/mL，其特异度能达到98.2%。Peptest是一种无创、快速检测胃蛋白酶的试剂盒，只需要收纳1 mL的口腔或气管分泌物，10 min后即可获得结果^[71-73]^。因此，该方法有望成为诊断误吸的主要方法和手段^[74-76]^。

## 8 处理和治疗

术中误吸的处理最关键的就是能够快速识别并做出反应。主要措施为：口咽部及气管内抽吸、体位、药物、机械通气。

### 8.1 抽吸

插管过程中观察到口咽部或气道内存有胃内容物，应立即使用负压吸引器抽吸干净，操作过程中，保持患者头部朝下并转向对侧，术前应明确患者是否存在有颈椎相关疾病。此外，可弯曲支气管镜和硬支气管镜可选择性使用。

### 8.2 体位

头低脚高的Trendelenburg体位，可利于内镜的检查和操作，同时，也易使误吸物从气道中流出，将危险降至更小。

### 8.3 血管活性药物

有研究^[77]^表明硝普钠在急性肺水肿时可通过扩张肺血管而抑制水肿肺泡的血管收缩。

### 8.4 激素的使用

化学性肺炎是否需要使用糖皮质激素一直存在有争议，临床研究数据也很有限。有报道使用激素可缓解肺部炎症反应，也有报道可以加重炎症反应^[78-80]^。而在经验性使用中发现，低剂量、短期激素使用可以改善患者的死亡率，而副反应并没有显著增加，相反，长期或是大剂量的使用可增加死亡的风险^[81-84]^。另一项前瞻性研究表明，使用激素治疗的患者中，肺损伤的改善比单纯使用安慰剂改善更快，但是，监护室的住院时长显著高于未使用激素的患者，同时，两组的并发症无统计学差异^[85, 86]^。

### 8.5 抗生素

通常咽喉水平以下的呼吸道是无菌的，微量胃酸误吸可由机体的正常廓清机制清除，不会发生肺炎，但受到胃酸损害的肺很容易并发细菌感染^[24]^。因此，大量吸入口咽部致病微生物可导致吸入性肺炎的发生，3%-26%的化学性肺炎患者在恢复期间可出现继发性肺部感染，一项回顾性研究发现，与单纯支持治疗相比，经验性使用抗生素并没有降低患者死亡率^[87, 88]^。因此，对于单纯化学性肺炎，一般不需要使用抗生素治疗，但如果误吸后48 h，临床症状未缓解，发热、白细胞增多、胸片提示渗出增多，需考虑使用抗生素^[55, 89, 90]^，48 h-72 h后应再次评估患者症状和体征，以决定是否需要继续使用，如果症状缓解，胸片提示侵润消失，则应该停止使用抗生素^[10]^。

### 8.6 肺表面活性物质(pulmonary surfactant, PS)

新生儿透明膜病中使用PS可显著提高肺内的气体交换，增加肺表面张力、对抗肺水肿，增强肺的顺应性^[91-93]^。Slater等^[94]^给患有卡氏肺囊虫病肺炎合并ARDS的患者使用PS治疗后，其肺泡动静脉氧分压差显著减低，连续使用2天后，第3天正常拔除气管插管，恢复良好。但目前仍缺乏临床随机对照试验进一步验证PS的疗效。

### 8.7 呼吸支持治疗

肺通气支持(SIMV, PEEP)和静脉补液(高分子量胶体)可为肺提供支持治疗。机械通气除取决于患者常规呼吸指标参数外，还取决误吸物体积的大小和未来向ARDS发展的趋势和程度。

严重误吸所致的心脏骤停，除立即心肺复苏，气管内插管，及时有效清除气管内误吸物外，其早期使用体外膜肺氧合(extracorporeal membrane oxygenation, ECMO)，不仅可以稳定患者循环，还可进一步评估心肺复苏后各种生命指标。而基于成人ARDS的研究发现，术中误吸继发ARDS的患者早期使用ECMO有益，但到目前为止，还没有关于ECMO在误吸所致的心脏骤停应用中的相关大型研究。

## 9 预防

### 9.1 术前危险因素评估

对于麻醉科和外科来说，对患者及其既往病史的全面了解至关重要，如体格检查和既往症状的全面系统回顾。根据美国麻醉医师协会的建议，麻醉前访视应包括诸如胃食管反流病、食管运动障碍、吞咽困难、糖尿病、胃排空延迟、食管部相关肿瘤等病史的询问^[95, 96]^。

### 9.2 术前禁食

一般来说，清液、乳制品和清淡膳食/非清液的禁食时间分别是2 h、4 h和6 h，这样可提高麻醉安全性、患者舒适性和机体代谢平衡，同时也可降低术中误吸发生的概率。与过去术前禁食10 h-12 h相比，缩短禁食时间可减少患者口渴、烦躁、紧张不适，甚至降低分解代谢和大大缩短住院时间^[97]^。长时间的禁食，对手术患者并没有益处，并且也不符合当前ERAS的理念^[98]^。然而，对于胸外科医师来说，有一点需要注意：对于麻醉风险增加的人，如考虑到有导致胃排空不良和增加固体食物残留的疾病，如食道旁疝气、失弛缓症和阻塞性食道癌等，可行几天的全液体饮食^[99, 100]^。

### 9.3 胃管置入

预先置入胃管可在一定程度上降低术中误吸发生的风险，但目前尚缺乏相关临床研究数据。急诊手术中误吸事件的发生率是普通手术的4.1倍，除非怀疑肠梗阻，并没有发现术前常规胃排空可减少误吸^[3]^。因此，鼻胃管的使用应由手术外科医生和麻醉师根据患者的情况和需要协同考虑。鼻胃管置入也有助于预防术中呕吐和随后的抽吸处理。

### 9.4 抑酸和促胃动力治疗

抑酸药可提高胃液pH值，减轻误吸所致的肺损伤和炎症反应，常用药物为质子泵抑制剂(p roton pump inhibitor, PPI)(兰索拉唑、埃索美拉唑、兰索拉唑、奥美拉唑、泮托拉唑和雷贝拉唑)，H_2_受体阻滞剂(西咪替丁、法莫替丁、尼扎替丁和雷尼替丁)^[101]^。多潘立酮、甲氧氯普胺等促胃动力药可通过促进胃排空而降低误吸风险。两者作用类似，但H_2_受体拮抗剂的疗效更为显著，同时服用两种药物则效果相似^[102, 103]^。Puig等^[104]^发现胃pH值低于2.5，胃容量高于25 mL时更易发生误吸。除胃液pH值外，胆汁对呼吸道也有损害作用。美国麻醉师协会未推荐常规使用上述药物来降低术中误吸的风险^[95]^。

### 9.5 体位

最佳体位可以降低误吸发生的概率。Takenaka等^[105]^发现头部向下倾斜，喉头与口保持水平，是防止吸入的必要条件。头部向下倾斜15°-20°，再加环状软骨压迫，是减少误吸的最佳体位。

## 10 结语

术中误吸具有潜在致命风险，能显著减慢患者术后的康复速度。因此，了解患者误吸相关危险因素和易感因素，努力降低有风险的操作以及发生误吸后立即采取有效处理措施，可降低误吸发生和优化误吸相关并发症结局。当前，ERAS理念已深入临床，并显示出显著的优越性，围手术期肺保护也已指南、专家共识等形式应用于临床，它们在降低围手术期并发症和缩短住院时间上确实提供了可行的实践指导方案。

但是，仍有许多问题亟待解决，术中误吸目前并没有一个明确的诊断标准和方法，仍需探索合适的方法和技术来解决。只有有了诊断标准，才能更加明确的指导临床实践。因此，探寻方便有效的诊断方法和预防措施仍旧是当前国内外ERAS的重要研究课题部分。不论是基础研究还是临床研究，都因遵循“以患者为中心”的医学理念进行开展，减少康复时间和住院费用，优化国家医保和医疗资源分配。

总之，深入研究术中误吸所致的肺脏病理改变及探索相关诊断标准是未来ERAS的基石，同时也是ERAS发展的动力，只有这样，ERAS才能在未来临床医疗实践中做到有理有据，推动临床继续向前发展。
